# Soluble Tumor Necrosis Factor Receptor 1 and 2 Predict Outcomes in Advanced Chronic Kidney Disease: A Prospective Cohort Study

**DOI:** 10.1371/journal.pone.0122073

**Published:** 2015-03-30

**Authors:** Nathalie Neirynck, Griet Glorieux, Eva Schepers, Francis Verbeke, Raymond Vanholder

**Affiliations:** Nephrology Section, Department of Internal Medicine, Ghent University Hospital, Ghent, Belgium; University of Florida, UNITED STATES

## Abstract

**Background:**

Soluble tumor necrosis factor receptors 1 (sTNFR1) and 2 (sTNFR2) have been associated to progression of renal failure, end stage renal disease and mortality in early stages of chronic kidney disease (CKD), mostly in the context of diabetic nephropathy. The predictive value of these markers in advanced stages of CKD irrespective of the specific causes of kidney disease has not yet been defined. In this study, the relationship between sTNFR1 and sTNFR2 and the risk for adverse cardiovascular events (CVE) and all-cause mortality was investigated in a population with CKD stage 4-5, not yet on dialysis, to minimize the confounding by renal function.

**Patients and methods:**

In 131 patients, CKD stage 4-5, sTNFR1, sTNFR2 were analysed for their association to a composite endpoint of all-cause mortality or first non-fatal CVE by univariate and multivariate Cox proportional hazards models. In the multivariate models, age, gender, CRP, eGFR and significant comorbidities were included as covariates.

**Results:**

During a median follow-up of 33 months, 40 events (30.5%) occurred of which 29 deaths (22.1%) and 11 (8.4%) first non-fatal CVE. In univariate analysis, the hazard ratios (HR) of sTNFR1 and sTNFR2 for negative outcome were 1.49 (95% confidence interval (CI): 1.28-1.75) and 1.13 (95% CI: 1.06-1.20) respectively. After adjustment for clinical covariables (age, CRP, diabetes and a history of cardiovascular disease) both sTNFRs remained independently associated to outcomes (HR: sTNFR1: 1.51, 95% CI: 1.30-1.77; sTNFR2: 1.13, 95% CI: 1.06-1.20). A subanalysis of the non-diabetic patients in the study population confirmed these findings, especially for sTNFR1.

**Conclusion:**

sTNFR1 and sTNFR2 are independently associated to all-cause mortality or an increased risk for cardiovascular events in advanced CKD irrespective of the cause of kidney disease.

## Introduction

Chronic kidney disease (CKD) has been linked to increased risk for cardiovascular disease and mortality independent of traditional cardiovascular risk factors [1]. This has at least in part been attributed to micro-inflammation, since inflammatory markers were associated to cardiovascular disease or mortality in different CKD cohorts not on dialysis [2–5] as well as on dialysis [6–8].

Soluble tumor necrosis factor receptor 1 (sTNFR1) and soluble tumor necrosis factor receptor 2 (sTNFR2) are the circulating forms of their membrane bound counterparts (mTNFR1 and mTNFR2) which are essential for tumor necrosis factor alpha (TNFα)-signalling via different pathways. Interaction between TNFα and both mTNFR leads to a pro-inflammatory stimulus via activation of nuclear factor kappa B (NF-κB) or activator protein 1 (AP-1), while only mTNFR1 contains a death domain through which signalling leads to apoptosis [9]. The soluble receptors are released into circulation via shedding of membrane receptors, in exosomes or via alternative splicing of mRNA transcripts which leads to a loss of the transmembrane and cytoplasmic domains [10].

In contrast to TNFα, which failed to be associated to mortality or cardiovascular events in CKD [2], both circulating receptors are potential biomarkers in chronic kidney disease as predictors for outcome, be it in selected populations with diabetic nephropathy [11–13] or in early or moderate CKD [12,14]. Whereas diabetes can be seen as a pro-inflammatory stimulus per se and a broad range of GFR implies a higher risk for confounding because of the known relationship of sTNFRs with kidney function, the association of both sTNFRs to mortality and cardiovascular events has not yet been evaluated in a CKD population that was not selected on a specific cause nor in patients who suffer from advanced stages of disease.

In the present study, we evaluated the predictive value of sTNFR1 and sTNFR2 for the risk for all-cause mortality or cardiovascular events in a population with advanced CKD (stage 4–5 not on dialysis). Evaluating outcome in advanced CKD minimizes the influence of eGFR on outcomes and on the concentration of the evaluated marker. This may allow to generate hypotheses on the contribution of other important mechanisms in the pathophysiology of CKD, such as inflammation, while reducing the impact of their intrinsic association to kidney function.

## Patients and Methods

### Ethics statement

This study was approved by the local Ethics Committee (Ethical Committee, Ghent University Hospital, Ghent, Belgium) and performed in accordance to the Declaration of Helsinki. Written informed consent was obtained from all participants.

### Study population

All non-transplanted CKD patients stage 4 and 5 not on dialysis, attending the Nephrology outpatient clinic and included in the biobank sample collection of the Nephrology Department of the Ghent University Hospital between January 2011 and August 2012, were included in this study (n = 131). Samples were processed immediately after collection and stored at—80°C. Outcomes were registered prospectively.

Baseline clinical parameters (age, gender, blood pressure, heart rate, height and weight) and etiology of the underlying kidney disease (vascular, diabetic nephropathy, glomerular disease/auto-immune, interstitial/postrenal, others and unknown) were registered. Body mass index (BMI) was calculated as weight/height^2^ (kg/m^2^), mean arterial pressure (MAP) as the sum of 1/3 of the systolic and 2/3 of the diastolic blood pressure and pulse pressure (PP) as the difference between systolic and diastolic blood pressure. Estimated glomerular filtration rate (eGFR) was calculated based on the creatinine-based CKD-EPI formula [15]. The following comorbidities were recorded: cardiovascular history when at least one of the following was present: arterial cardiovascular disease (coronary, cerebral or peripheral), atrial fibrillation or heart failure (requiring hospitalisation); malignancy; diabetes mellitus, defined as a history of diabetes or treatment with insulin or oral antidiabetic drugs; hypertension, defined as current hypertension (>140/90 mmHg) or the use of antihypertensive drugs; hypercholesterolemia, defined as history of elevated serum cholesterol or treatment with lipid lowering drugs and smoking status (active versus no/former smoker). The start of renal replacement therapy (RRT) during follow-up was registered.

Patients were followed and adverse cardiovascular events (CVE), defined as acute coronary syndrome (ACS), coronary artery bypass graft (CABG), percutaneous transluminal coronary angioplasty (PTCA) and stroke, and all-cause mortality were registered until September 19^th^ 2014. The composite endpoint in this study was all-cause mortality or a first non-fatal CVE, whichever occurred first. The collection of the events was done by a single researcher and was based on investigation of the hospital patient file.

### Measurements

The concentrations of sTNFR1, sTNFR2 and TNFα, were determined on plasma samples by ELISA (R&D Systems, Abingdon, United Kingdom). Serum creatinine, C-reactive protein (CRP) and albuminemia were measured with routine laboratory methods.

### Statistical analysis

Continuous data are expressed as mean with standard deviation or median with interquartile range depending on their distribution, and analysed by Student’s t-test or Mann Whitney-test as appropriate. Binary categorical data are expressed as frequencies and analysed with chi-square test. Linear regression analysis was performed between sTNFR1 and sTNFR2 as dependent variables and the different clinical parameters as independent variables.

Univariate and multivariate analyses were performed by using Cox proportional hazards models to estimate the relationship between sTNFR1 and sTNFR2, as continuous variables and the risk for negative outcome defined as the composite endpoint of all-cause mortality or the occurrence of a first non-fatal CVE. Univariate analysis was also performed to assess the association between the baseline clinical variables and outcome.

In multivariate analysis, separate models were built for sTNFR1 and sTNFR2. Due to collinearity, sTNFR1 and sTNFR2 were not entered together in a model. First, age and gender were forced into a model with sTNFR1 or sTNFR2 (model 1). In the second model, possible confounders, which correlated significantly with sTNFR1 or sTNFR2, i.e. eGFR, CRP and TNFα (the latter only for sTNFR2), were added to model 1. The third model included the clinical covariables which reached a significance of p < 0.05 with outcome in univariate analysis (age, CRP, history of cardiovascular disease and diabetes mellitus). In a separate analysis in the group of patients for which albumin concentrations were available (n = 73), albumin was also included as covariate in the model. Model 2 and 3 were analysed by forward and backward regression procedures based on the likelihood ratio test and gave similar results. In this publication, only the results of the stepwise forward procedure are reported as hazard ratio (HR) with a 95% confidence interval (CI).

The analysis described above was repeated in a subgroup analysis of patients with and without diabetes.

A p-value <0.05 was considered as statistically significant.

All statistical analyses were performed with SPSS Statistics V22.0 (SPSS Inc., Chicago, IL, USA) for Windows (Microsoft Corp, Redmond, WA, USA).

## Results

### Baseline clinical characteristics

In this study population including 131 patients with CKD stage 4–5 not on dialysis, the etiology of the underlying kidney disease was distributed as follows: renal vascular disease, mainly nephrangiosclerosis, n = 37 (28.2%); diabetic nephropathy, n = 28 (21.4%); glomerular/auto-immune disease, n = 20 (15.3%); interstitial/postrenal, n = 15 (11.5%); other, mainly autosomal dominant polycystic kidney disease, (unilateral) nephrectomy or use of calcineurin inhibitors in liver or heart transplants, n = 23 (17.5%); and unknown n = 8 (6.1%). Forty events (30.5%), defined as the composite endpoint of all-cause mortality or first non-fatal CVE, occurred after a total median follow-up of 33 months [interquartile range 24–39 months]. Twenty-nine patients (22.1%) died (cardiovascular (n = 3), malignancy (n = 3), infection (n = 7), ESRD (n = 1), unknown (n = 15)) and 11 (8.4%) had a first non-fatal CVE (ACS (n = 1), PTCA (n = 4), CABG (n = 2), stroke (n = 4)), of which 5 patients died subsequently during follow-up. Seven events occurred after the start of RRT, which was initiated in 35 patients during follow-up. Baseline clinical characteristics and biochemical parameters of the entire population and those with and without event are presented in Table 1. Patients who reached the composite endpoint, were older, had a higher CRP, lower albumin and had more often a history of cardiovascular disease or diabetes. There was no difference in eGFR between both groups, complying with one of the aims of this study, i.e. to restrict the influence of kidney function on outcome among subgroups by selecting a patient population with an eGFR over a narrow range. Both sTNFRs were significantly higher in the group who had an event.

**Table 1:** Baseline characteristics of the entire study population and according to the occurrence or not of the studied events.

Variable	Entire cohort	Death or first CVE		
		No	Yes	p-value
	N = 131	N = 91	N = 40	
Age (years)	73 [62–89]	70 [58–77]	78 [69–83]	<0.001
Gender (M)	83 (63.4)	56 (61.5)	27 (67.5)	0.51
BMI (kg/m^2^)	28.3 ± 5.7	28.2 ± 5.0	28.4 ± 7.1	0.83
MAP (mmHg)	99 ± 13	100 ± 13	99 ± 14	0.84
PP (mmHg)	61 ± 19	60 ± 18	65 ± 20	0.10
HR (/min)	69 ± 13	69 ± 13	72 ± 14	0.29
eGFR (ml/min/1.73m^2^)	22.8 [16.1–27.0]	23.0 [16.6–27.5]	21.2[15.5–26.4]	0.40
CVD	64 (48.1)	38 (41.8)	26 (65.0)	0.02
DM	51 (38.9)	29 (31.9)	22 (55.0)	0.02
Malignancy	32 (24.4)	19 (20.9)	13 (32.5)	0.15
Cholesterol	90 (68.7)	60 (65.9)	30 (75.0)	0.30
AHT	108 (82.4)	75 (82.4)	33 (82.5)	0.99
Smoking (active)	12 (9.1)	9 (10.5)	3 (7.9)	0.66
Start RRT during FU	35 (26.7)	27 (29.7)	8 (20.0)	0.25
Albumin (g/dl)	4.0 ± 0.9	4.2 ± 0.8	3.7 ± 1.0	<0.01
CRP (mg/l)	3.0 [1.0–8.0]	2.0 [0.9–4.0]	8.0 [2.3–12.0]	<0.001
TNFα (pg/ml)	4.6 [3.7–6.0]	4.6 [3.3–5.5]	4.9 [3.8–6.8]	0.13
sTNFR1 (ng/ml)	4.0 [3.1–5.1]	3.8 [2.8–4.8]	4.7 [3.9–6.0]	<0.001
sTNFR2 (ng/ml)	7.4 [5.8–9.4]	6.9 [5.2–8.8]	7.9 [6.4–12.0]	<0.001

Data are presented as means ± standard deviation or medians with interquartile range between square brackets. For binary variables, frequencies with percentages between brackets are given. CVE: cardiovascular event, N = number of patients, M: male, BMI: body mass index, MAP: mean arterial pressure, PP: pulse pressure, HR: heart rate, eGFR: estimated glomerular filtration rate, CVD: history of cardiovascular disease, DM: diabetes mellitus, cholesterol: hypercholesterolemia, AHT: arterial hypertension, RRT: renal replacement therapy, FU: follow-up, CRP: C-reactive protein, TNFα: tumor necrosis factor alpha, sTNFR1: soluble tumor necrosis factor receptor 1, sTNFR2: soluble tumor necrosis factor receptor 2.

**Fig 1:**
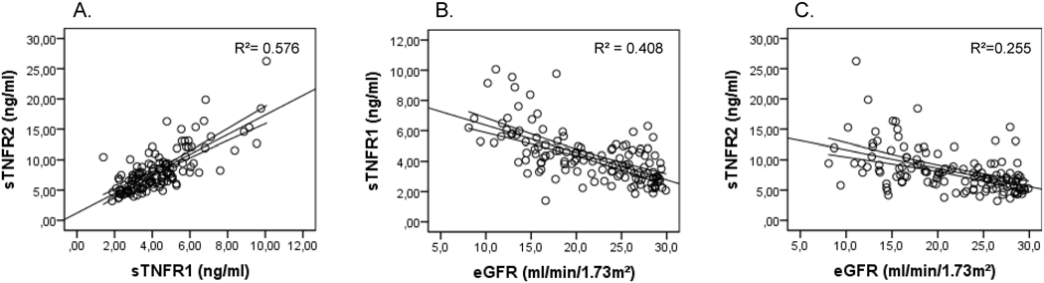
Dot plots showing the association between sTNFR1 and sTNFR2 (panel A), eGFR and sTNFR1 (panel B) and sTNFR2 (panel C). sTNFR1: soluble tumor necrosis factor receptor 1. sTNFR2: soluble tumor necrosis factor 2. eGFR: estimated glomerular filtration rate. R^2^: coefficient of determination. The straight line represents the best fitted linear regression line with 95% confidence interval for the mean.

sTNFR1 and sTNFR2 were strongly correlated (r = 0.76, p < 0.001) (Fig. 1). Linear regression analysis with sTNFR1 and sTNFR2 as dependent variables showed only a correlation with eGFR (sTNFR1: r = -0.64, p <0.001; sTNFR2: r = -0.50, p < 0.001), CRP (sTNFR1: r = 0.52, p < 0.001; sTNFR2: r = 0.54, p < 0.001) and albumin (sTNFR1: r = -0.39, p < 0.05; sTNFR2: r = -0.39, p<0.05). TNFα was only moderately correlated to sTNFR2 (r = 0.29, p < 0.01) and not to sTNFR1 (r = 0.17, p = 0.059). No significant correlations were found between both sTNFR and clinical variables listed in Table 1.

### sTNFR1, sTNFR2 and outcome (death or first non-fatal CVE)

**Fig 2:**
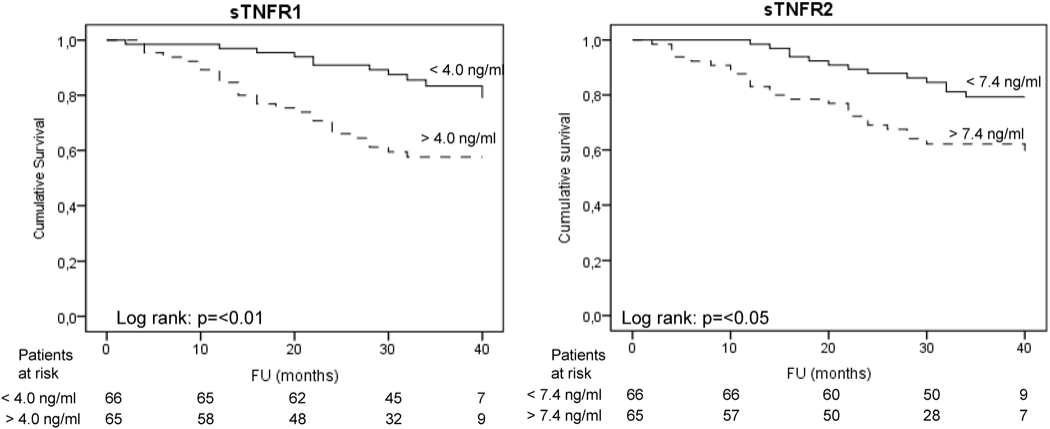
Kaplan Meier survival plots for sTNFR1 > or < than the median (4.0 ng/ml) and sTNFR2 > or < than the median (7.4 ng/ml). sTNFR1: soluble tumor necrosis factor receptor 1. sTNFR2: soluble tumor necrosis factor 2.

In univariate Cox proportional hazards analysis, sTNFR1 [Hazard ratio (HR): 1.49, 95% confidence interval (CI): 1.28–1.75] and sTNFR2 [HR: 1.13, 95% CI: 1.06–1.20] were associated to adverse outcomes. Older age, a higher CRP, a lower albumin concentration, a history of cardiovascular disease and diabetes mellitus also showed an association (Table 2). Kaplan Meier survival curves for both receptors are depicted in Fig. 2. For both sTNFRs, concentrations above the median were associated with higher event rates.

**Table 2:** Univariate Cox proportional hazards analysis for outcome (death or first adverse cardiovascular event).

Variable	B	HR [95% CI]
**sTNFR1 (per ng/ml)**	**0.402**	**1.49 [1.28–1.75]** ***
**sTNFR2 (per ng/ml)**	**0.118**	**1.13 [1.06–1.20]** ***
TNFα (per pg/ml)	0.024	1.02 [0.96–1.09]
**CRP (per mg/l)**	**0.017**	**1.02 [1.01–1.03]** ***
Gender (M)	0.316	1.37 [0.71–2.68]
**Age (per year)**	**0.058**	**1.06 [1.03–1.09]** ***
eGFR (per ml/min/1.73m^2^)	-0.025	0.98 [0.93–1.03]
MAP (per mmHg)	-0.001	1.00 [0.98–1.02]
PP (per mmHg)	0.014	1.01 [1.00–1.03]
Albumin (per g/dl)	0.036	0.71 [0.52–0.98]
BMI	-0.001	1.00 [0.95–1.06]
**CVD**	**0.757**	**2.13 [1.11–4.08]** *
**DM**	**0.774**	**2.17 [1.16–4.04]** *
Malignancy	0.480	1.62 [0.83–3.14]
AHT	-0.052	0.95 [0.42–2.15]
Cholesterol	0.304	1.36 [0.66–2.78]
Smoking	-0.209	0.81 [0.25–2.64]

HR: Hazard ratio, CI: confidence interval. In bold, variables with p-value < 0.05, included in the multivariate model (Table 3, model 3).

*: p < 0.05.

***: p < 0.001.

sTNFR1: soluble tumor necrosis factor receptor 1, sTNFR2: soluble tumor necrosis factor receptor 2, TNFα: tumor necrosis factor alpha, CRP: C-reactive protein, eGFR: estimated glomerular filtration rate, MAP: mean arterial pressure, PP: pulse pressure, BMI: body mass index, CVD: history of cardiovascular disease, DM: diabetes mellitus, AHT: arterial hypertension, cholesterol: hypercholesterolemia.

In multivariate models, sTNFR1 and to a smaller extent sTNFR2 remained significantly associated to outcome. In the first model (Table 3A) after adjustment for age and gender, both sTNFRs had an increased HR of 1.52, 95% CI 1.30–1.77 (sTNFR1) and 1.07, 95% CI 1.03–1.21 (sTNFR2). When further adjustments were made for possible confounders which were correlated to the sTNFRs in linear regression analysis (eGFR, CRP, TNFα), sTNFR1 and sTNFR2 together with age remained independently associated to outcome (Table 3B). In the third model after adjustment for age, CRP, a history of cardiovascular disease and diabetes mellitus, both receptors remained independently associated to outcome, the hazards ratio for sTNFR1 (HR: 1.51, 95% CI: 1.30–1.77) again being higher than for sTNFR2 (HR: 1.13, 95% CI: 1.06–1.20). Age was the only significant covariate in the model with sTNFR1, while in the model with sTNFR2, age and diabetes mellitus were significant covariables (Table 3C).

**Table 3:** Multivariate Cox proportional hazards models for death or first CVE.

Variable	B	HR [95% CI]	p-value
**A. Model 1: sTNFR1 or sTNFR2, age, gender**			
A1. sTNFR1			
sTNFR1 (per ng/ml)	0.417	1.52 [1.30–1.77]	< 0.001
age (per year)	0.065	1.07 [1.03–1.11]	< 0.001
gender (M)			n.s.
A2. sTNFR2			
sTNFR2 (per ng/ml)	0.135	1.14 [1.07–1.22]	< 0.001
age (per year)	0.065	1.07 [1.03–1.10]	< 0.001
gender (M)			n.s.
**B. Model 2: sTNFR1 or sTNFR2, age, gender, eGFR, CRP, TNFα**			
B1. sTNFR1			
sTNFR1 (per ng/ml)	0.414	1.51 [1.30–1.77]	<0.001
age (per year)	0.063	1.07 [1.03–1.10]	<0.01
gender, CRP, eGFR			n.s.
B2. sTNFR2			
sTNFR2 (per pg/ml)	0.136	1.15 [1.08–1.22]	<0.001
age	0.057	1.06 [1.02–1.10]	<0.01
gender, CRP, eGFR, TNFα			n.s.
**C. Model 3: sTNFR1 or sTNFR2, age, CRP, CVD, DM**			
C1. sTNFR1			
sTNFR1 (per ng/ml)	0.414	1.51 [1.30–1.77]	<0.001
age (per year)	0.063	1.07 [1.03–1.10]	<0.001
CRP, DM, CVD			n.s.
C2. sTNFR2			
sTNFR2 (per ng/ml)	0.119	1.13 [1.06–1.20]	<0.001
age (per year)	0.063	1.07 [1.03–1.10]	<0.001
DM	0.644	1.90 [1.02–3.56]	<0.05
CRP, CVD			n.s.

CVE: cardiovascular event, HR: Hazard ratio, CI: confidence interval. In model 2: TNFα, only included in model with sTNFR2. sTNFR1: soluble tumor necrosis factor receptor 1, sTNFR2: soluble tumor necrosis factor receptor 2, TNFα: tumor necrosis factor alpha, eGFR: estimated glomerular filtration rate, CRP: C-reactive protein, TNFα: tumor necrosis factor alpha, CVD: history of cardiovascular disease, DM: diabetes mellitus, n.s.: not significant.

When including also albumin in this multivariate model, sTNFR1 (HR: 1.46, 95%CI: 1.16–1.84) remained significantly associated to adverse outcome, while sTNFR2 was not. Albumin itself was not a significant covariate.

In summary, after adjustment for significant clinical covariables, sTNFR1 and sTNFR2 remained independently associated to increased risk of death or cardiovascular events. This association was stronger for sTNFR1 than for sTNFR2 (Table 3).

### Subgroup analysis: patients without and with diabetes

Eighteen of the eighty patients without diabetes had an adverse event (22.5%). Patients, having an event, were older (80 vs. 68.5 years, p < 0.001), had a higher CRP (9.0 vs. 2.0 mg/l, p < 0.001), had more often a history of malignancy (50% vs. 19.4%, p < 0.01) and were less likely to start RRT during FU (5.6% (n = 1) vs. 29% (n = 18), p < 0.05). There were no differences for the other clinical variables listed in Table 1 (S1 Table). In univariate analysis sTNFR1 and sTNFR2 were significantly associated to adverse outcome, as well as age, CRP and malignancy (S2 Table). After adjustment for age, CRP and malignancy, only sTNFR1 remained significant in the model with a HR of 1.85, 95% CI 1.38–2.47 (Table 4).

**Table 4:** Multivariate Cox proportional hazards model for death or first CVE in the subgroup of non diabetic patients.

Variable	B	HR [95% CI]	p-value
**Full model with sTNFR1**			
sTNFR1 (per ng/ml)	0.615	1.85 [1.38–2.47]	<0.001
age (per year)	0.082	1.09 [1.03–1.15]	<0.01
CRP, malignancy			n.s.
**Full model with sTNFR2**			
CRP (per mg/l)	0.039	1.04 [1.02–1.06]	<0.001
age (per year)	0.065	1.07 [1.01–1.13]	<0.05
sTNFR2 (per ng/ml), malignancy			n.s.

CVE: cardiovascular event, HR: Hazard ratio, CI: confidence interval, sTNFR1: soluble tumor necrosis factor receptor 1, sTNFR2: soluble tumor necrosis factor receptor 2, TNFα: tumor necrosis factor alpha, eGFR: estimated glomerular filtration rate, CRP: C-reactive protein, TNFα: tumor necrosis factor alpha, n.s.: not significant.

In the subgroup of diabetes patients (n = 51), only sTNFR1 and not sTNFR2 concentrations were significantly higher in the patients reaching the composite endpoint (4.5 vs. 3.8 ng/ml, p < 0.05) (S3 Table). In univariate analysis both receptors were associated to adverse outcome (S4 Table). This significant association persisted only for sTNFR1 after adjustment for age, gender, CRP and eGFR in multivariate analysis (HR: 1.35, 95% CI, 1.11–1.65).

## Discussion

This study evaluated the value of sTNFR1 and sTNFR2 as biomarkers for their association to the composite endpoint of all-cause mortality or first non-fatal cardiovascular event in a cohort with advanced CKD irrespective of the underlying etiology. The main finding of this study is that sTNFR1 and sTNFR2 are associated to adverse outcome, even after adjustment for clinical covariables, such as age, gender, eGFR, CRP. The association with outcome was stronger for sTNFR1 than for sTNFR2. In the subgroups of patients without and with diabetes only sTNFR1 was after adjustment associated to outcome.

To the best of our knowledge, this is the first study to investigate associations between sTNFR1 and sTNFR2 and adverse outcomes in advanced CKD irrespective of the etiology. The study may be particularly relevant since by design, the narrow range of eGFR (<30 ml/min/1.73m^2^) minimized the impact of eGFR on outcome, which was confirmed by the lack of association to adverse outcomes (Tables 2 and 3) and there is also no influence of dialysis therapy. In spite of the small eGFR range, we found a relatively good correlation between eGFR and sTNFR1 (r = -0.64) and sTNFR2 (r = -0.50), underscoring that sTNFR1 and sTNFR2 with respective molecular weights of approximately 30 and 40 kDa, are probably mainly eliminated by renal clearance [16–18].

Also, in large cohort studies in the general population, sTNFR1 [19,20] and sTNFR2 [19,21] have been associated to an increased risk for major cardiovascular events [19,21] or mortality [20], even after adjustment for traditional risk factors [21]. However, in these cohorts, and in contrast to our study (Table 3), no corrections were made for kidney function [21] or the association was only found when creatinine clearance ranged up to 75 ml/min, which implies a much broader range of eGFR than in the present study [19]. As a consequence, it cannot be excluded that in these studies the sTNFR concentrations rather reflected the association of eGFR with adverse outcomes than that of sTNFRs itself. In the present study, sTNFRs were more informative for adverse outcome than what could be explained through their correlation with eGFR alone, since associations of sTNFRs to adverse outcome remained significant after adjustment for eGFR, while eGFR was not significant (Table 3). This observation could strengthen the hypothesis that sTNFRs are also markers for underlying pathophysiological mechanisms of cardiovascular risk in CKD. This is supported by findings in other populations with increased cardiovascular risk. sTNFR1 at admission for acute myocardial infarction was associated to infarct size and worse left ventricular function 4 months post-infarction [22], to the composite endpoint of death and new onset heart failure [23] and to long term cardiac and all-cause mortality post-infarction even when corrected for relevant covariates such as creatinine clearance [24].

This is also consistent with the increased mortality risk associated to sTNFR1 in patients with diabetic nephropathy [12,13]. In the present study, the association between sTNFRs and outcome was also confirmed in the subgroup of patients without diabetes (Table 4), be it more strongly for sTNFR1 (in univariate and multivariate analysis) than for sTNFR2 (only in univariate analysis). In diabetic patients, only sTNFR1 was associated to adverse outcome.

Remarkably, although linked to each other in the same inflammatory TNFα–pathway, sTNFR1 and sTNFR2 do not entirely yield the same information, despite the strong mutual correlation between both receptors as observed in our study (r = 0.76) and also reported previously [11]. sTNFR1 did not correlate to TNFα, while a significant but modest correlation was found for sTNFR2 (r = 0.29). In the subgroups of patients without or with diabetes only sTNFR1 and not sTNFR2 was associated to outcome in multivariate analysis. This could point towards different mechanisms of generation and elimination of sTNFR1, sTNFR2 and TNFα, independently of each other. The pathophysiological processes behind their presence in circulation in CKD are incompletely understood and need further investigation in order to explain these different associations with outcome.

Constitutively, both sTNFRs are released from cell membranes by shedding [18,25] or as full-length receptors in exosomes [26,27]; the full-length sTNFR1 in exosomes being the most abundant form of sTNFR1 in serum of healthy controls [27]. Both forms are capable of binding TNFα [18,27]. Following acute pro-inflammatory stimuli, shedding is intensified, resulting in an increase in circulating sTNFR concentration [25,28] and in a possible modulation of immune response [29]. The function of these soluble receptors is debated: they are inhibitors for TNFα, especially in acute inflammatory settings [18,30], but, in more chronic conditions and in proportion to their concentration, they also increase the half-life of TNFα and may act as slow-release reservoirs of TNFα, enhancing its cytotoxicity [31]. Which process prevails in CKD still needs to be elucidated, although the sTNFR concentration range (3–12 ng/ml) for which Aderka et al. [31] found a prolonged activity of TNFα function, corresponds to concentrations found in CKD. Together with our findings of a relationship with adverse outcome, this could indicate that sTNFRs in CKD would rather increase the negative effects of TNFα than act as TNFα-inhibitors.

The limitations of the study are: the results cannot be generalized to all CKD stages, since we restricted the evaluation to CKD stage 4 and 5 intentionally to evaluate both receptors in a narrow range of eGFR, which also resulted in a rather small population. Furthermore, based on our results it is not yet possible to draw conclusions on the potential use or incremental value of these markers in clinical situations. Nevertheless, the study unravelled convincing correlations between sTNFRs and outcomes even after adjustment for confounders such as eGFR or diabetes. Hence our data underscore the relationship of TNFRs, with hard clinical outcomes irrespective of underlying kidney disease and the presence or absence of diabetes. The fact that especially sTNFR1 was consistently associated to different outcomes in advanced CKD as shown in this study as well as in community-based and specific high risk populations such as post-myocardial infarction, offers a strong argument in favour for its further evaluation in larger CKD cohorts.

## Supporting Information

S1 Table Baseline clinical characteristics in the subpopulation without diabetes. Data are presented as means ± standard deviation or medians with interquartile range between square brackets. For binary variables, frequencies with percentages between brackets are given. MACE: major adverse cardiovascular event, N = number of patients, M: male, BMI: body mass index, MAP: mean arterial pressure, PP: pulse pressure, HR: heart rate, eGFR: estimated glomerular filtration rate, CVD: history of cardiovascular disease, DM: diabetes mellitus, AHT: arterial hypertension, RRT: start of renal replacement therapy during follow-up, CRP: C-reactive protein, TNFα: tumor necrosis factor alpha, sTNFR1: soluble tumor necrosis factor receptor 1, sTNFR2: soluble tumor necrosis factor receptor 2.
(DOC)

S2 Table Univariate Cox proportional hazards analysis for outcome (death or first major adverse cardiovascular event) in the subpopulation without diabetes (n = 80). HR: Hazard ratio, CI: confidence interval. In bold, variables with p-value < 0.05, included in the multivariate model. sTNFR1: soluble tumor necrosis factor receptor 1, sTNFR2: soluble tumor necrosis factor receptor 2, TNFα: tumor necrosis factor alpha, CRP: C-reactive protein, eGFR: estimated glomerular filtration rate, MAP: mean arterial pressure, PP: pulse pressure, BMI: body mass index, CVD: history of cardiovascular disease, DM: diabetes mellitus, AHT: arterial hypertension.
(DOC)

S3 Table Baseline clinical characteristics in the subpopulation without diabetes. Data are presented as means ± standard deviation or medians with interquartile range between square brackets. For binary variables, frequencies with percentages between brackets are given. MACE: major adverse cardiovascular event, N = number of patients, M: male, BMI: body mass index, MAP: mean arterial pressure, PP: pulse pressure, HR: heart rate, eGFR: estimated glomerular filtration rate, CVD: history of cardiovascular disease, DM: diabetes mellitus, AHT: arterial hypertension, RRT: start of renal replacement therapy during follow-up, CRP: C-reactive protein, TNFα: tumor necrosis factor alpha, sTNFR1: soluble tumor necrosis factor receptor 1, sTNFR2: soluble tumor necrosis factor receptor 2
(DOC)

S4 Table Univariate Cox proportional hazards analysis for outcome (death or first major adverse cardiovascular event) in the subpopulation with diabetes (n = 51). HR: Hazard ratio, CI: confidence interval. In bold, variables with p-value < 0.05, included in the multivariate model. sTNFR1: soluble tumor necrosis factor receptor 1, sTNFR2: soluble tumor necrosis factor receptor 2, TNFα: tumor necrosis factor alpha, CRP: C-reactive protein, eGFR: estimated glomerular filtration rate, MAP: mean arterial pressure, PP: pulse pressure, BMI: body mass index, CVD: history of cardiovascular disease, DM: diabetes mellitus, AHT: arterial hypertension.
(DOC)
