# Supplementary material for: Soluble Tumor Necrosis Factor Receptor 1 and 2 Predict Outcomes in Advanced Chronic Kidney Disease: A Prospective Cohort Study
Source: PLoS One. 2015 Mar 30;10(3):e0122073. doi: 10.1371/journal.pone.0122073 (PMC4379033; doi:10.1371/journal.pone.0122073)
Supplement: S1 Table — Data are presented as means ± standard deviation or medians with interquartile range between square brackets. For binary variables, frequencies with percentages between brackets are given. MACE: major adverse cardiovascular event, N = number of patients, M: male, BMI: body mass index, MAP: mean arterial pressure, PP: pulse pressure, HR: heart rate, eGFR: estimated glomerular filtration rate, CVD: history of cardiovascular disease, DM: diabetes mellitus, AHT: arterial hypertension, RRT: start of renal replacement therapy during follow-up, CRP: C-reactive protein, TNFα: tumor necrosis factor alpha, sTNFR1: soluble tumor necrosis factor receptor 1, sTNFR2: soluble tumor necrosis factor receptor 2. (DOC) [file pone.0122073.s001.doc]

**S1 Table. Baseline clinical characteristics in the subpopulation without diabetes**

| Variable | Population | No event | Death/first MACE | p-value |
| --- | --- | --- | --- | --- |
|  | N=80 | N= 62 | N= 18 (22.5%) |  |
| **Age (years)** | **72 [61-80.5]** | **68.5 [50.0-75.3]** | **80 [75.5-84]** | **<0.001** |
| Gender (M) n(%) | 52 (65) | 38 (61) | 14 (78) | 0.18 |
| BMI (kg/m²) | 27.8 ± 5.1 | 28.1 ± 5.3 | 27.2 ± 4.4 | 0.36 |
| MAP (mmHg) | 101 ±14 | 100 ± 14 | 104 ± 14 | 0.35 |
| PP (mmHg) | 60 ± 18 | 59 ± 17 | 64 ± 20 | 0.31 |
| P (/min) | 69 ± 6 | 68 ±12 | 71 ±12 | 0.53 |
| eGFR (ml/min/1.73m²) | 24.1 [16.6-27.8] | 23.9 [16.6-27.9] | 25.0 [15.4-26.9] | 0.60 |
| CVD | 34 (42.5) | 24 (38.7) | 10 (2.6) | 0.20 |
| **Malignancy** | **21 (26.3)** | **12 (19.4)** | **9 (50.0)** | **<0.01** |
| Hypercholesterolemia | 41 (61.3) | 36 (58.1) | 13 (72.2) | 0.28 |
| AHT | 60 (75) | 47 (75.8) | 13 (72.2) | 0.76 |
| Smoking (yes) | 8 (10.7) | 7 (12.3) | 1 (5.6) | 0.42 |
| Albuminemia (g/dl) (n=46) | 4.1 ± 0.8 | 4.2 ± 0.8 | 3.9 ± 0.6 | 0.35 |
| TNFα (pg/ml) | 4.6 [3.5-5.9] | 4.6 [3.3-5.2] | 4.9[3.7-7.8] | 0.23 |
| **sTNFR1 (pg/ml)** | **4.0 [2.94-5.1]** | **3.8 [2.8-4.8]** | **4.8 [4.1-5.9]** | **<0.001** |
| **sTNFR2 (pg/ml)** | **7.4 [5.7-9.4]** | **6.9 [5.2-9.2]** | **7.2 [9.4-12.3]** | **<0.01** |
| **CRP (mg/l)** | **2.0 [1.0-6.0]** | **2.0 [0.8-4.1]** | **9.0 [2.8-34.8]** | **<0.001** |
| **RRT** | **19 (23.8)** | **18 (29.0)** | **1 (5.6)** | **<0.05** |
