# Supplementary material for: Soluble Tumor Necrosis Factor Receptor 1 and 2 Predict Outcomes in Advanced Chronic Kidney Disease: A Prospective Cohort Study
Source: PLoS One. 2015 Mar 30;10(3):e0122073. doi: 10.1371/journal.pone.0122073 (PMC4379033; doi:10.1371/journal.pone.0122073)
Supplement: S2 Table — HR: Hazard ratio, CI: confidence interval. In bold, variables with p-value < 0.05, included in the multivariate model. sTNFR1: soluble tumor necrosis factor receptor 1, sTNFR2: soluble tumor necrosis factor receptor 2, TNFα: tumor necrosis factor alpha, CRP: C-reactive protein, eGFR: estimated glomerular filtration rate, MAP: mean arterial pressure, PP: pulse pressure, BMI: body mass index, CVD: history of cardiovascular disease, DM: diabetes mellitus, AHT: arterial hypertension. (DOC) [file pone.0122073.s002.doc]

**S2 Table. Univariate Cox proportional hazards analysis for outcome (death or first major adverse cardiovascular event) in the subpopulation without diabetes (n=80)**

| Variable | B | HR [95% CI] | P |
| --- | --- | --- | --- |
| **sTNFR1 ( per ng/ml)** | **0.578** | **1.78 [1.35-2.36]** | **<0.001** |
| **sTNFR2 (per ng/ml)** | **0.192** | **1.21 [1.06-1.38]** | **<0.01** |
| TNFα ( per pg/ml) | 0.045 | 1.05 [0.95-1.16] | 0.37 |
| **CRP (per mg/l)** | **0.050** | **1.05 [1.03-1.07]** | **<0.001** |
| Gender (M) | 0.781 | 2.18 [0.72-6.64] | 0.17 |
| **Age (per year)** | **0.082** | **1.09 [1.03-1.15]** | **<0.01** |
| eGFR (per ml/min/1.73m²) | -0.014 | 0.99 [0.92-1.06] | 0.72 |
| PP (per mmHg) | 0.012 | 1.01 [0.99-1.04] | 0.32 |
| MAP (per mmHg) | 0.017 | 1.02 [0.98-1.05] | 0.33 |
| Albuminemia (per g/dl) | -0.240 | 0.79 [0.44-1.41] | 0.42 |
| CVD (yes) | 0.527 | 1.69 [0.67-4.29] | 0.27 |
| Malignancy (yes) | 1.170 | 3.22 [1.28-8.14] | 0.01 |
| AHT (yes) | -0.180 | 0.83 [0.30-2.33] | 0.72 |
| Hypercholestelemia (yes) | 0.575 | 1.78 [0.63-4.99] | 0.28 |
| BMI (per kg/m²) | -0.029 | 0.97 [0.89-1.07] | 0.54 |
| Smoking (yes) | -0.694 | 0.50 [0.07-3.75] | 0.50 |
