# Supplementary material for: Soluble Tumor Necrosis Factor Receptor 1 and 2 Predict Outcomes in Advanced Chronic Kidney Disease: A Prospective Cohort Study
Source: PLoS One. 2015 Mar 30;10(3):e0122073. doi: 10.1371/journal.pone.0122073 (PMC4379033; doi:10.1371/journal.pone.0122073)
Supplement: S3 Table — Data are presented as means ± standard deviation or medians with interquartile range between square brackets. For binary variables, frequencies with percentages between brackets are given. MACE: major adverse cardiovascular event, N = number of patients, M: male, BMI: body mass index, MAP: mean arterial pressure, PP: pulse pressure, HR: heart rate, eGFR: estimated glomerular filtration rate, CVD: history of cardiovascular disease, DM: diabetes mellitus, AHT: arterial hypertension, RRT: start of renal replacement therapy during follow-up, CRP: C-reactive protein, TNFα: tumor necrosis factor alpha, sTNFR1: soluble tumor necrosis factor receptor 1, sTNFR2: soluble tumor necrosis factor receptor 2 (DOC) [file pone.0122073.s003.doc]

**S3 Table. Baseline clinical characteristics in the subpopulation without diabetes**

| Variable | Population | No event | Death/first MACE | p-value |
| --- | --- | --- | --- | --- |
|  | N = 51 | N = 29 | N = 22 |  |
| Age (years) | 74 [61-81] | 73 [61-79] | 78.5 [68-82.9] | 0.12 |
| Gender (M) n(%) | 31 (60.8) | 118(62.1) | 13 (59.1) | 0.83 |
| BMI (kg/m²) | 28.9 ± 6.5 | 28.5 ± 4.5 | 29.4 ± 8.6 | 0.62 |
| MAP (mmHg) | 97 ± 11.7 | 98.4 ± 11.6 | 95.1 ± 11.8 | 0.40 |
| PP (mmHg) | 63.5 ± 19.9 | 61.3±18.0 | 66.5 ± 22.3 | 0.43 |
| P (/min) | 70.8 ± 14.6 | 69.6 ± 13.3 | 72.1 ± 16.2 | 0.44 |
| eGFR (ml/min/1.73m²) | 20.7 [15.9-25.5] | 20.7 [15.1-25.7] | 20.4 [15.5-25.9] | 0.74 |
| CVD | 30 (58.8) | 14 (48.3) | 16 (72.7) | 0.08 |
| Malignancy | 11(21.6) | 7 (24.1) | 4 (18.2) | 0.61 |
| Hyperholesterolemia | 41 (80.4) | 24 (82.8) | 17 (77.3) | 0.63 |
| AHT | 48 (94.1) | 28 (96.6) | 20 (90.9) | 0.40 |
| Smoking (yes) | 4 (8.2) | 2 (6.9) | 2 (10.0) | 0.70 |
| Albuminemia (g/dl) (n=27) | 3.9 ± 1.0 | 4.2 ± 0.7 | 3.6 ± 1.1 | 0.09 |
| TNFα (pg/ml) | 4.4 [3.8-6.1] | 4.4 [3.3-5.7] | 4.9 [3.8-6.8] | 0.37 |
| **sTNFR1 (pg/ml)** | **4.0 [3.3-4.9]** | **3.8 [2.9-4.6]** | **4.6 [3.6-6.2]** | **<0.05** |
| sTNFR2 (pg/ml) | 7.1 [5.8-8.9] | 7.0 [5.4-8.7] | 7.5 [6.2-11.9] | 0.19 |
| **CRP (mg/l)** | **4.0 [1.0-9.0]** | **2.0 [0.75-6.0]** | **8.0 [2.0-12.25]** | **<0.05** |
| RRT | 16 (31.4) | 9 (31.0) | 7 (31.8) | 0.95 |
