# Supplementary material for: Soluble Tumor Necrosis Factor Receptor 1 and 2 Predict Outcomes in Advanced Chronic Kidney Disease: A Prospective Cohort Study
Source: PLoS One. 2015 Mar 30;10(3):e0122073. doi: 10.1371/journal.pone.0122073 (PMC4379033; doi:10.1371/journal.pone.0122073)
Supplement: S4 Table — HR: Hazard ratio, CI: confidence interval. In bold, variables with p-value < 0.05, included in the multivariate model. sTNFR1: soluble tumor necrosis factor receptor 1, sTNFR2: soluble tumor necrosis factor receptor 2, TNFα: tumor necrosis factor alpha, CRP: C-reactive protein, eGFR: estimated glomerular filtration rate, MAP: mean arterial pressure, PP: pulse pressure, BMI: body mass index, CVD: history of cardiovascular disease, DM: diabetes mellitus, AHT: arterial hypertension. (DOC) [file pone.0122073.s004.doc]

**S4 Table. Univariate Cox proportional hazards analysis for outcome (death or first major adverse cardiovascular event) in the subpopulation with diabetes (n= 51)**

| Variable | B | HR [95% CI] | P |
| --- | --- | --- | --- |
| **sTNFR1 (per ng/ml)** | **0.300** | **1.35 [1.11-1.65]** | **<0.01** |
| **sTNFR2 (per ng/ml)** | **0.087** | **1.09 [1.01-1.18]** | **<0.05** |
| TNFα (per pg/ml) | 0.000 | 1.00 [0.92-1.09] | 1.00 |
| CRP (per mg/l) | 0.009 | 1.01 [1.00-1.02] | 0.09 |
| Albuminemia (per g/dl) | -0.332 | 0.72 [0.48-1.07] | 0.10 |
| Gender (M) | -0.034 | 0.97 [0.41-2.27] | 0.94 |
| Age (per year) | 0.036 | 1.04 [0.99-1.08] | 0.11 |
| eGFR (per ml/min/1.73m²) | -0.032 | 0.97 [0.90-1.05] | 0.41 |
| PP (per mmHg) | 0.015 | 1.01 [0.99-1.04] | 0.24 |
| MAP (per mmHg) | -0.011 | 0.99 [0.95-1.03] | 0.59 |
| HR (per min) | 0.023 | 1.02 [0.19 -1.06] | 0.16 |
| CVD | -0.664 | 0.42 [0.16 -1.08] | 0.07 |
| Malignancy | -0.255 | 0.77 [0.26-2.30] | 0.65 |
| AHT | -1.579 | 0.21 [0.47-0.91] | 0.04 |
| Hypercholerolemia | -0.610 | 0.54 [0.20-1.51] | 0.24 |
| BMI | 0.003 | 1.00 [0.94-1.07] | 0.93 |
| smoking | 0.126 | 1.14 [0.26-5.05] | 0.87 |
